# Shared Decision-Making in Growth Hormone Therapy—Implications for Patient Care

**DOI:** 10.3389/fendo.2018.00688

**Published:** 2018-11-22

**Authors:** Carlo L. Acerini, David Segal, Sherwin Criseno, Kei Takasawa, Navid Nedjatian, Sebastian Röhrich, Mohamad Maghnie

**Affiliations:** ^1^Department of Paediatrics, University of Cambridge, Cambridge, United Kingdom; ^2^Department of Paediatrics, University of the Witwatersrand, Johannesburg, South Africa; ^3^Department of Endocrinology, University Hospitals Birmingham NHS Foundation Trust, Birmingham, United Kingdom; ^4^Department of Paediatrics and Developmental Biology, Tokyo Medical and Dental University, Tokyo, Japan; ^5^Novo Nordisk Health Care AG, Zurich, Switzerland; ^6^Department of Paediatrics, IRCCS Istituto Giannina Gaslini, University of Genova, Genova, Italy

**Keywords:** growth hormone therapy, delivery device, shared decision-making, patient autonomy, treatment adherences

## Abstract

Several studies have shown that adherence to growth hormone therapy (GHT) is not optimal. There are several reasons why patients may not fully adhere to their treatment regimen and this may have implications on treatment success, patient outcomes and healthcare spending and resourcing. A change in healthcare practices, from a physician paternalistic to a more patient autonomous approach to healthcare, has encouraged a greater onus on a shared decision-making (SDM) process whereby patients are actively encouraged to participate in their own healthcare decisions. There is growing evidence to suggest that SDM may facilitate patient adherence to GHT. Improved adherence to therapy in this way may consequently positively impact treatment outcomes for patients. Whilst SDM is widely regarded as a healthcare imperative, there is little guidance on how it should be best implemented. Despite this, there are many opportunities for the implementation of SDM during the treatment journey of a patient with a GH-related disorder. Barriers to the successful practice of SDM within the clinic may include poor patient education surrounding their condition and treatment options, limited healthcare professional time, lack of support from clinics to use SDM, and healthcare resourcing restrictions. Here we discuss the opportunities for the implementation of SDM and the barriers that challenge its effective use within the clinic. We also review some of the potential solutions to overcome these challenges that may prove key to effective patient participation in treatment decisions. Encouraging a sense of empowerment for patients will ultimately enhance treatment adherence and improve clinical outcomes in GHT.

## Growth hormone therapy (GHT) and adherence to treatment is not optimal

The treatment success of many chronic conditions including those associated with the need for long-term GHT, may be dependent upon several factors including patient characteristics, therapy area challenges, treatment access, and healthcare provisions. These factors also vary widely in different countries. One key contributor to treatment success however is patient adherence to their treatment regimen and commitment to long-term therapy. The World Health Organization defines treatment adherence as “the extent to which a person's behaviour—taking medication, following a diet, and/or executing lifestyle changes—corresponds with agreed recommendation from a healthcare provider” (1). In the context of GHT, this may involve patient adherence with respect to timing of the growth hormone (GH) dose, GH dose frequency, and method of administration (device instructions) (2).

The definition of adherence however may also be extended to reflect an active and continuous process that comprises more than just treatment posology and dose frequency, but rather adherence to a whole treatment plan as born out of a decision process between both a patient and their clinician. It is also important to consider the notion of persistence to treatment. Persistence may be defined as “the duration of time from initiation to discontinuation of therapy” (2). When considering GHT, a patient's treatment journey may be lengthy and both patients and their families must commit to a long-term process. Clinical outcomes will therefore not only be impacted by patient adherence to their treatment regimen, but also by how well they persist with their treatment over a long period. Thus, it is important to clearly distinguish between these two definitions when considering clinical outcomes and patient commitment to their treatment plan.

When considering patient adherence and persistence to GHT, one might place more importance on what happens when patients do *not* adhere or persist with their treatment regimens. Non-adherence to therapy is of considerable concern and may have implications for treatment outcomes (2, 3). A report in 2003 by the World Health Organization reported that treatment adherence in patients with chronic diseases is as low as ~50% (1). In the case of GHT specifically, non-adherence has been shown to impact linear growth and growth velocity (4, 5). Poor adherence to therapy may also ultimately incur increased healthcare service utilisation and greater healthcare spending (5–7).

There are a number of studies that demonstrate that adherence to GHT is not optimal, however the exact rates of non-adherence reported differ considerably (5, 8–14). A study by Oyarzabal et al. reported “excellent” compliance in 74.0% of the 473 patients surveyed, while a study by Rosenfeld et al., reported a similar proportion (64–77%) of *non*-compliance following their survey of GHT patients. Variation in definitions of non-adherence (fixed cut-offs or any action that interferes with an individual's treatment plan) (15, 16), patient populations and study methodology may contribute to these differences. Despite this, there appears to be a consensus that adherence to GHT could be improved.

Adherence to GHT may be monitored in several ways including prescription monitoring, patient/parent questionnaires/interviews, and biomarker testing (17, 18). Monitoring of issued, renewed or encashed GHT prescriptions offers perhaps the most objective measure of adherence, however it does not provide details regarding specific days of treatment missed or timing inconsistencies, but rather only a gross measure of patient adherence (19). Conversely, patient questionnaires and interviews may offer a more thorough account of patient non-adherence but are often limited by the potential for misrepresentation of information, given patients difficulty to recall past events or fear of disappointing the doctor (20). Urinary GH levels (21, 22) or biomarker monitoring of serum insulin-like growth factor 1 (IGF-1) (23, 24), may also be an option to monitor adherence. However, challenges associated with timing of monitoring, data variation, and assay variability/reliability make these methods difficult to implement effectively and accurately (20). IGF-1 monitoring is yet to be validated as a biomarker for adherence given its uncertainty as to whether low levels represent poor adherence, low sensitivity to GHT or other factors (24). Finally, injection devices with incorporated electronic technologies capable of monitoring and recording information on injections can be of assistance, if they are accepted and used by patients and their caregivers (25). Choosing a suitable means by which to monitor adherence will ultimately be driven by low-cost, user-friendliness, and practicality of application (20, 26).

There are many reasons why patients may not fully adhere to their GHT regimen and may be broadly categorised into treatment-specific, convenience, cognitive, and personal factors. Treatment-specific factors may include the appearance of unwanted side effects, apparent ineffectiveness of medication, and access to treatment. Convenience challenges including the logistics of GH administration, cold chain preservation, travel, and difficulties with the dosing regimen may also impact adherence to treatment. Cognitive factors may include forgetfulness or poor education regarding why treatment is necessary, while personal factors including patient-doctor relationships, fear of needles, poor support, social pressure, and denial may also contribute to non-adherence in patients (14, 17, 27). A study by Bagnasco et al., in children and adolescents receiving GHT in 46 paediatric centres across Italy, found that the most frequently reported reasons for missing GH doses were being away from home, forgetfulness, not feeling well and pain (28). Finally, adherence to GHT may also be influenced by whether GHT is self-injected by the patient or administered by their parent/caregiver. Available data are currently somewhat conflicting and more studies are needed to investigate how patient or parent-led administration of GHT may impact adherence rates and ultimately treatment outcomes for patients (9, 13, 28, 29). Importantly, consideration of adherence as an “all or none” process may be too simplistic; the causes of non-adherence may not always be predictable, nor may they always be modifiable. There will most likely be a time, place or situation-dependent variable to some of these factors, meaning that overall, the concept of adherence is highly complex.

Understanding the ways in which patients may be better encouraged to actively participate in their treatment journey however, may serve to promote patient engagement and avoid non-adherence in the first instance.

## Introduction to shared decision-making (SDM)

SDM may be defined as “an approach where clinicians and patients share the best available evidence when faced with the task of making decisions, and where patients are supported to consider options, to achieve informed preferences” (30). The concept of SDM was first established in 1982 (31) and originates predominantly from the notion of patient-centred care (32) and a rebalance of the onus of decision-making between clinician and patient. This is accompanied by a switch from a focus on beneficence to a focus on patient autonomy and supports a culture of active patient participation in decisions to undertake therapy and their involvement in the details of their treatment regimen (33, 34). Beneficence and non-maleficence refer to a paternalistic approach whereby patient interests are maintained, if need be, without patient participation (35). Patient autonomy however upholds a patient's right to make decisions regarding their medical care without the undue influence of their healthcare provider (30). While extreme ends of a physician paternalistic/patient autonomous spectrum are subject to criticism, establishing a balance between the two may well serve to satisfy the best interest of the patient. Indeed, SDM offers a means by which this balance can be achieved and encourages an active participation of patients in their own healthcare in partnership with their doctor (36). Current guidelines from the National Institute for Health and Care Excellence (NICE) and the position statement of British Society for Paediatric Endocrinology and Diabetes (BSPED) on the choice of delivery device for GH prescribing (37), discuss the need for implementation of SDM in medical practice and detail the steps doctors should take to implement active patient involvement in their treatment (38). In addition, there is growing evidence that SDM improves patient adherence and treatment outcomes in several therapy areas, not limited to GHT (28, 39–43).

## Challenges and benefits of implementing SDM in GH disorders

When considering SDM in the context of GH disorders, one must consider the unique challenges that a predominantly paediatric population of patients encounter. The notion of a balance between physician paternalistic and patient autonomous healthcare decisions is further complicated by the presence of a patient's parent/guardian as a third party who may wish to participate in SDM. Some parents do not wish to be involved in a SDM process and prefer a wholly paternalistic approach by the clinician, while others wish to take an active role in the decision-making process (44). SDM cannot therefore be a “one size fits all” paradigm that may be applied in all situations. Rather a careful consideration must be made of the opportunities for SDM and the benefits this may have for both parent and patient. Very young patients are unable to make treatment decisions themselves and as such, parent preferences will likely determine treatment decisions for the patient. As patients get older, their involvement in the decision-making process will likely increase and the practice of SDM should again be reviewed and adapted to best suit their needs and treatment goals. Capturing both patient and parent needs as part of this decision-making process will encourage involvement in the treatment journey and positively impact adherence to GHT as a result.

While the addition of parents or caregivers to the patient/doctor dyad may prove challenging to manage, the implementation of effective SDM may help patients and/or parents to understand the importance of not missing doses and the overall value of GHT. Adherence to GHT may be affected by an underestimation of the consequences of missed GH injections. In a study by Rosenfeld and Bakker, negative influencers of adherence to GHT included a perception that missed GHT doses did not have serious implications (13). While missed insulin injections for patients with type-1 diabetes may have both a serious and almost immediate impact on the patient, patients who miss GHT injections may not notice any immediate changes and may associate their GHT with a lack of effectiveness (17, 45). While research has confirmed a correlation between poor adherence to GHT and sub-optimal patient outcomes (5), there remains a need to educate patients and/or parents in the importance of regular GHT dosing and the consequences should injections be missed.

There may also be a lack of appreciation, among patients and/or parents, of the benefits of GHT beyond just increased height. Whilst height velocity/linear growth are the most commonly assessed measures of treatment success in prepubertal patients, the benefits of GHT extend beyond improved growth. GHT is known to have roles in body composition, lipid metabolism, androgen action, myocardial function, and cardiovascular health, as well as, improved quality-of-life perception (46). Benefits of GHT in patients with GH-related disorders other than GH deficiency (GHD) have also been demonstrated (47–57). While improvement in adult height is widely considered the main motivation for treatment in children born small for gestational age (SGA), GHT is associated with improved body composition, blood pressure, lipid metabolism and reduced fat mass (52, 53). Improved psychosocial functioning, quality of life, and intelligence are also noted benefits of GHT in children born SGA (54, 55). Similarly, in addition to increased final height, GHT has also been associated with improvements in bone age and body composition in patients with Noonan syndrome (51) and improvements in lean body mass and reduced adiposity in Turner's syndrome (50). Finally, in patients with Prader-Willi syndrome, improved visuospatial functioning, cognition, and motor activity have been reported following treatment with GHT (47, 56, 57). Awareness of the effects of GHT, beyond just linear growth may thus serve to positively influence adherence to GHT given the wider-reaching health benefits afforded to patients (58).

Considering the variety of potential benefits afforded by GHT and the specificities of its effect in different growth-related disorders, it is vital that SDM is appropriately tailored to the specific GH disorder being treated. Patients/parents must be adequately educated in the potential benefits of GHT, such that expectations of therapy can be accordingly managed. SDM will thus support patient/parent education and encourage a discussion of the wider benefits of GHT, specific to the patient's diagnosis. In conjunction with this, awareness of the quality of evidence collected for certain patient-reported outcomes (as commonly assessed with small-scale qualitative patient surveys/interviews, observational studies etc.) must also guide SDM appropriately such that doctors may be entirely transparent in their disease education.

SDM may go some way to address these knowledge gaps around missed doses and the wider benefits of GHT by encouraging patients to take an active and informed role in their therapy. Opportunities for SDM during a patient's treatment journey include the decision to begin treatment, which GH delivery device to use, if and how to self-monitor adherence and growth progress, goal-setting, when to review treatment plans following transition to adolescence and whether to continue or terminate treatment. Opportunities to facilitate SDM at every stage may not be possible, however the goal of SDM is not necessarily to allow patients to dictate every aspect of their treatment plan but rather take an active role in their own care and in doing so encourage a greater “ownership” and satisfaction with their treatment.

In line with the notion that SDM cannot be arbitrarily implemented in every case, country-specific variations in practice of SDM also need to be considered. For example, limited access to certain treatment devices in some countries, as dictated by cost restrictions or regulatory approvals, may mean there is little room for SDM in the context of device choice compared with the options that may be available to patients in other healthcare systems with greater resources. Despite this, SDM is still crucial in other points of a patient's treatment journey, including patient goal setting, motivations, and commitment to therapy. In Japan, more importance is placed on within-family discussions than on prolonged discussions with the doctor, encouraging the active participation of family elders when making decisions regarding treatment strategies (39). In other healthcare settings, including those in the United Kingdom (UK), clinician time may be a barrier to SDM given challenges to staff resource and predefined consultations times (59). In the UK especially, the role of specialist endocrine nurses and/or advanced nurse practitioners as a support to a patient's journey with GHT is of crucial importance and will offer the counselling, training, and education that is required when patients first make the decision to undergo GHT (60, 61). Finally, in other countries including Italy, there is a need for a standardised and accredited process for the effective implementation of SDM, to allow for a patient-specific care plan whereby patients are appropriately educated about their condition and the treatment options available such that treatment adherence may be encouraged (28).

Defining a prescriptive model of SDM may therefore be limiting. However, a form of SDM based on specific cases, cultures, and healthcare system resourcing may prove to be of greater utility. Below, we discuss some of the opportunities for SDM during a patient's treatment journey in more detail.

### SDM and the decision to start GHT

When considering SDM during a patient's treatment journey, it must be recognised that the decision to initiate therapy is likely to be the first opportunity where it can be implemented. For GHT, as with most medical strategies, there may be more than one potential treatment path including delaying treatment initiation or even not pursuing treatment at all (32). Each path will be associated with varying therapeutic impact and side effects. It is therefore critical for doctors to work with patients and/or parents to help them understand their personal goals and preferences so that they come to an informed and shared decision regarding whether to begin treatment or not (32). In some healthcare settings, this decision may also be driven, or at least influenced by the cost of therapy (particularly when considering the long-term nature of this treatment). SDM may therefore be adjusted so as to support consideration of a patient's therapy options, within the confines of a specific healthcare costing system.

Following initiation of GHT, patients are likely to remain on their treatment regimen for many years (62), during which time their preferences, motivations, and support structures are likely to change significantly (17). As such, fully engaging patients during their initial treatment decisions may prove vital in nurturing patient “buy-in” further down the line. Values clarification and preference elicitation exercises may be particularly useful for early treatment decisions. Values clarification encourages patients to consider those aspects of the treatment option that are most and least important to them while preference elicitation may encourage patients to consider which of their options are most favoured, and conversely, least favoured (63).

Finally, the decision to begin GHT may be a crucial point at which to introduce SDM to set a precedent for a patient's involvement and engagement in their own subsequent treatment plan. Patients may be unaware that they have a role to play in decisions about their medical care (64, 65). While some may not wish to be fully involved in the process of SDM, reassuring patients that the decision to begin GHT is theirs and that they have several options available to them, may help to garner patient engagement with their healthcare plan and to introduce them to the options that are available.

### SDM and selection of GH injection device (a typical example)

The decision regarding which injection device to use is another example of a stage during treatment that may be aided by the active participation of both patient/parent and doctor to decide on the most appropriate treatment choice. Historically, doctors and nursing staff made the choice of device type on behalf of their patient, however patients are now encouraged to participate in the process of device choice prior to treatment. Indeed, current NICE guidelines state that “the choice of product should be made on an individual basis after informed discussion between the responsible clinician and the patient and/or their carer and the advantages and disadvantages of the products available, taking into consideration therapeutic need and the likelihood of adherence to treatment” (62).

Likewise, the position statement of the BSPED on the Choice of Delivery Device for Growth Hormone Prescribing, recommends against “automatic substitution (dispensing one brand instead of another equivalent or interchangeable brand) by professionals other than the prescribing team, without consultation with the hospital consultant managing the growth disorder” (37). Patient choice will thus most likely feature in this consultation with the clinician when considering device change. Whilst there is little evidence to predict which factors may most likely drive patient device selection (12), there is growing opinion that injection pen devices that increase the convenience of GH administration are favoured by patients and may subsequently go some way to improve adherence to treatment (66). In a web-based survey assessing the impact of storage flexibility on the daily life of patients and caregivers administering GH, 86% of respondents who currently used a refrigerator-only product stated that they would “prefer” or “strongly prefer” to use a storage-flexible product. In the same survey, the proportion of respondents who reported never missing an injection was significantly greater for respondents using a storage-flexible product (76%) compared with those using a refrigeration-only product (57% *p* < 0.05) (67).

A study by Gau et al. reported that patient involvement in device selection may improve adherence beyond the influence of device features (39). This longitudinal group comparison study of 46 patients with GHD investigated the influence of patient choice on adherence to, and the therapeutic effects of GHT over 3 years, using a questionnaire survey. Eighteen participants were not given choice of device and assigned to a specific GH device whilst the remaining 28 were given device choice (*n* = 13 of which chose the same device as the first group). Patient choice improved adherence to GHT and resulted in improved therapeutic effects (linear growth and IGF-1 levels) compared with those patients who received no opportunity for device choice, even for those patients who chose the same device as patients with no choice. This study suggests that involving patients in the process of device choice can improve adherence to GHT and patient outcomes, independent of the device features (39).

As discussed previously, the cost of GHT devices may also be an important factor when making decisions and may be considered during SDM. Limitations due to healthcare budgets do not prevent the successful implementation of SDM to choose the most appropriate device out of those that are available. Even in cases where only one device may be available, this may still be facilitated by SDM, as there are multiple factors that are still appropriate to discuss with patients, including implementing the device into daily routine and monitoring of adherence. Finally, patient satisfaction of device may still drive device choice, rather than cost. Meinhardt et al. reported that patients attribute a high monetary value to specific device features that will enhance their treatment experience and together suggest a willingness to pay for such devices, that supersedes cost considerations (68).

Overall, while device features may have a considerable impact on patient adherence, for patients in a country where device features cannot be chosen (e.g., because of limited access or marketing authorisation), it is still possible to have acceptable treatment outcomes with a potentially “sub-optimal” GH delivery device. In such situations, patient commitment to their treatment regimen is crucial. Indeed, the notion of improved ownership of treatment may begin with an active involvement of patients when choosing their device such that the process of taking responsibility for treatment decisions actively encourages adherence and satisfaction. In summary, SDM, whether implemented at device choice, or at other points of a patient journey, appears to encourage a patient empowerment in their own treatment regimen with a potential for greater commitment to long-term treatment.

### SDM and the transition from paediatric to adolescent/young adult care

Transition may be defined as the “purposeful, planned movement of adolescents with chronic medical conditions from child-centred to adult-oriented health care” (69). However, in a number of chronic conditions, including GH disorders, a large proportion of patients terminate their therapy during their transition from paediatric to adolescent/young adult care (70). In a study of UK patients with congenital adrenal hyperplasia diagnosed during the neonatal period, only 10% of patients continued onto treatment in adult specialist endocrine centres (71). Reasons for termination of treatment may include insufficient knowledge of the treatment options available, a knowledge gap in the disease process and the benefits of GHT into adulthood, patient unwillingness to continue with their treatment regimen and patient hesitation to engage with a new healthcare team as part of their transition (70). Additionally, the cost of continued treatment may further influence a patient's decision to continue treatment into adulthood.

The effects of GHT vary widely depending on what stage of development it is administered. GHT in children normalises linear growth and helps patients achieve adult height within their genetic target range (70). In contrast, when continued into adolescence and adulthood in the case of persistent GHD, GHT helps to promote lean body mass, muscle strength and maintain metabolism and lipid profile (72). For this reason, the transition stage during a patient's treatment journey is an important opportunity for re-evaluation, review, and importantly, for SDM.

Transition into adolescence considered from a medical perspective requires a re-evaluation of the patient need for GHT, assessment of the potential for dose adjustments and review of the current treatment regimen (72). However, in addition to these considerations, the psychological, social, and educational needs of transitioning patients with chronic endocrine conditions must also be carefully considered (73, 74). Optimising adolescent transition such that patients garner a desire for their own autonomy (and indeed that parents relinquish that autonomy) may prove challenging (75). A position statement of the Canadian Paediatric Society recommends that preparation for transition should begin early in patients to more smoothly accommodate the transition process. While paediatric care is very much family-focused and patient parents/caregivers play a predominant role in treatment decisions as the first point of contact with the doctor, adult care is patient-focused and requires an autonomous approach in the absence of many interdisciplinary resources (76). Gradually increasing patient responsibility and education as patients get older will be best supported with SDM and encourage a collaborative effort between paediatric and adult endocrinologists, the patient and their family (76).

## Barriers to the implementation of SDM

**Figure 1:**
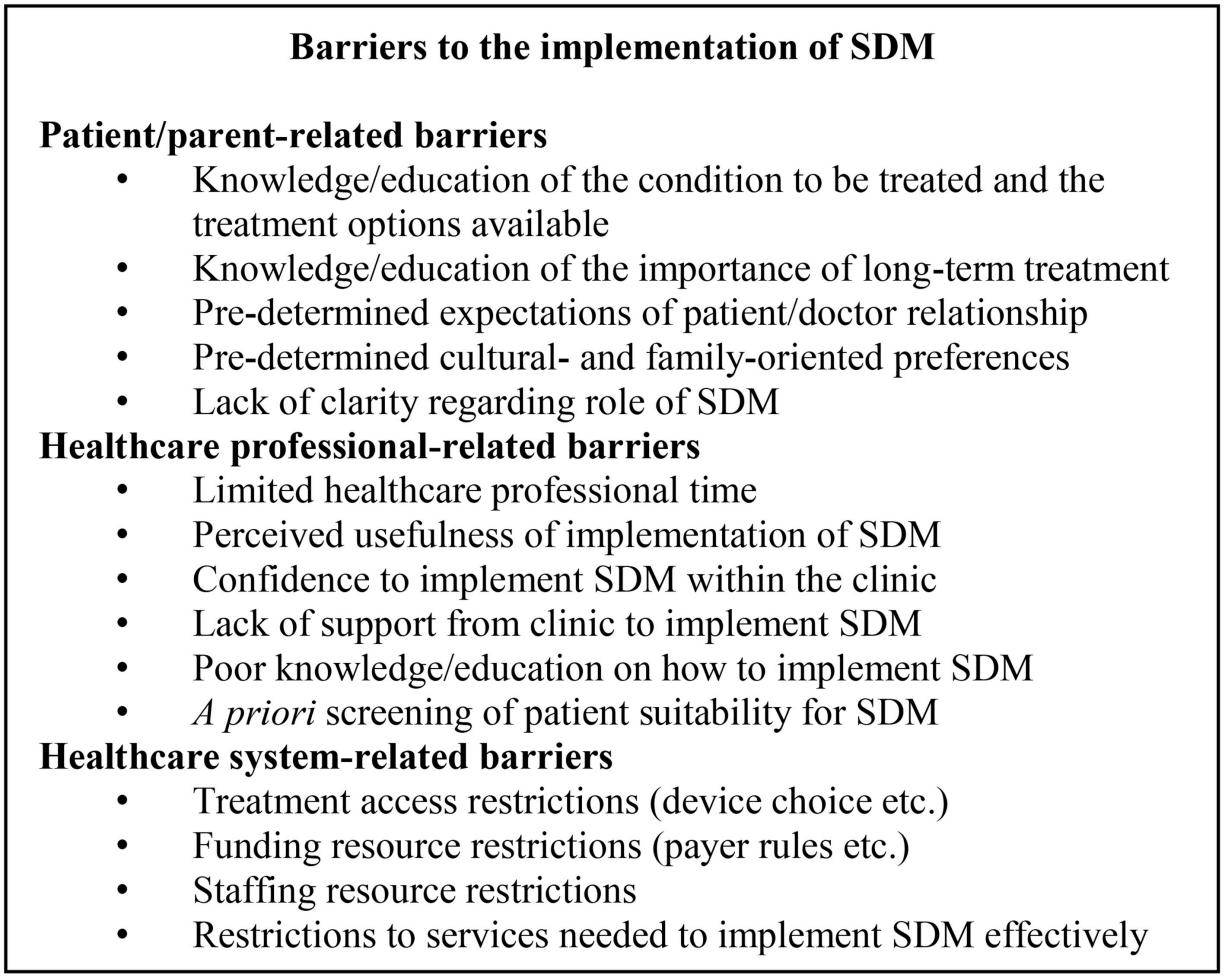
Barriers to the implementation of SDM. SDM, shared decision-making.

There are many barriers that may hinder the effective implementation of authentic SDM and may be categorised according to patient/parent-related barriers, healthcare professional-related barriers and healthcare system-related barriers (Figure 1). Understanding the challenges to SDM will serve to better inform the practice of its implementation and to increase awareness of some of the obstacles to overcome.

### Patient/parent-related barriers

Poor educational background may be a key barrier to the effective implementation of SDM. Authentic, patient-centred decision-making can only be achieved with an appropriate level of knowledge relating to the condition to be treated, the treatment options available and the importance of treating the condition. Patients may feel overwhelmed by a large volume of material detailing their treatment options. Not understanding why long-term treatment is necessary, or which device to choose may also serve to compromise authentic SDM. Moreover, this educational need should be applied to a patient's family; where patients may be too young to make a decision, the responsibility will fall with their parents/caregiver. There is somewhat conflicting evidence regarding whether parent education may have implications on patient adherence to their GHT. While there are some reports of no correlation between the level of parent education and adherence in patients (14), Rosenfeld et al., found that parents of highly GHT-compliant children had a lower education level (13). Conversely, another study noted a significant correlation between a GHT patient's father's educational level and adherence to their GHT (77). Whilst the data may not be consistent, patient/parent education may still be a crucial barrier to authentic SDM, engagement with, and ultimately adherence to GHT.

In addition, patient/parent-related barriers may include challenges that arise from an expectation of the roles of a doctor/patient relationship (36). Discrepancies between these expectations (some patients like to be told which decision is right for them given the expertise of the doctor) may make authentic SDM implementation difficult. Moreover, cultural, and family-determined preferences may drive some of these expectations and values. Indeed, in the context of a patient/doctor relationship, a study by Suurmond and Seeleman, noted that differing values regarding health and illness, role expectations and pre-established prejudices may compromise authentic SDM implementation (78). Similarly, patterns of parental preference when deciding their children's treatment decisions may further complicate authentic SDM. A study by Singh et al. reported that parents may place greater emphasis on different factors when considering their child's GHT. While the greatest proportion (36%) of the group promoted a risk-conscious approach to decision-making, others preferred a child-focused, cost-focused or device ease-of-use oriented preference (79). Additionally, in a survey of 69 parents with children treated with GHT, 79% of the group reflected that support from other parents and their doctor would be welcomed when considering GHT for their children (80). Recognising parent preferences is thus crucial to effective SDM. Parent-specific decision drivers and anxieties about their children's treatment may represent a significant barrier to effective SDM (79).

A study that investigated the incidence of GHT in children with idiopathic short stature, based on files collected at a tertiary paediatric medical centre in Israel, reported that boys accounted for a significantly larger proportion (65.8%) of the study group despite no significant differences between sexes with respect to age, height, body mass index or pubertal status. The similar characteristics of both groups would suggest there is no medically-determined reason for differences in GHT between sexes. However, the male predominance to receive GHT may reflect a family preference to treat boys with short stature more commonly than girls (81). Similar gender bias has also been reported in the United States (82) and Australia (83). While these studies need to be accompanied by a more thorough investigation of the psychological aspects of decisions to begin therapy, these data may implicate gender-based preferences of families as a barrier to authentic SDM given the possibility of pre-determined attitudes toward GHT.

Finally, in a study exploring patient-doctor interviews and perceptions of SDM, a lack of clarity of the concept of SDM was noted with patients regarding who would make the “final” decision. Confusion surrounding the role of SDM and the respective roles of the patient and doctor led to uncertain/tentative decisions from patients (84).

While evidence for these patient/parent-related barriers is largely derived from only qualitative patient surveys, there are commonly repeated references to outcomes that indicate that a lack of patient/parent education and pre-determined attitudes regarding GHT's value are crucial barriers to effective SDM.

### Healthcare professional-related barriers

Several studies have also investigated healthcare professional-related barriers that may hinder the effective implementation of SDM (78, 84–86). Limited healthcare professional time is perhaps the most commonly cited barrier and may be accompanied by a challenging window of opportunity in which to build an adequate patient-doctor relationship and authentically implement SDM (85). Contact time with patients and family is critical for SDM, however limited resources may be an obstacle to this. While there is a great deal of literature identifying time as a major concern for SDM (85), more recent evidence to evaluate the time needed for effective SDM is somewhat conflicting. Two studies trialling SDM initiatives for patients did not report increased consultation times compared with consultation without SDM, suggesting that time need not be a barrier to patient choice (87, 88).

Other cited barriers related to healthcare professionals may include their attitudes to SDM, perceived usefulness of its implementation within the clinic, lack of sufficient information, and self-confidence to implement it in practice, and cultural differences (85, 86). Some healthcare professionals have also cited that SDM may not be applicable given patient character or clinical situation and may suggest a degree of doctor *a priori* screening of the eligibility of SDM in a particular situation. The potential for misunderstanding of patient needs/desire for SDM thus makes this a concern that must be overcome (89). Additionally, a report by Cuttler and Silvers (90) noted that primary care of GHT is inconsistent given that doctors may vary considerably in their referral of short children to a paediatric endocrinologist. Moreover, endocrinologists may vary in their decisions to initiate GHT treatment in children with GH disorders. This variation may be driven by patient indicators including growth pattern and average height, or by family concern and their desire to seek medical treatment (91). Importantly however, this variation may also be driven by healthcare professional beliefs about the impact of short stature and the perception of the efficacy of GHT, particularly in children with less severe growth impairment (91). Together, pre-determined attitudes toward GHT may further threaten authentic SDM. While the notion of SDM in practice is widely accepted as a preferable method over that of a wholly paternalistic approach, there may be a discord between this acceptance and its implementation within the clinic.

### Healthcare system-related barriers

Healthcare rules may be a barrier to SDM in GHT, particularly in the context of treatment access and most specifically, device choice. Restricted resourcing may introduce a degree of bias associated with doctor advice and may compromise authentic SDM with patients; cost considerations and choice of the cheapest device may skew SDM. Resource restrictions like this will naturally vary in their impact depending on the healthcare system evaluated. A study by Chapman et al. investigated the drivers of GHT prescriptions in England with respect to price and patient preferences. This study reported only a weak correlation between the prescription and price of GHT in both primary and secondary care. GHT pricing was not the predominant driver in the prescription of GH but rather the ease of use and the number of steps required to prepare the GH dose. Whilst restricted resourcing of healthcare systems may be a barrier to effective SDM, this may not necessarily dissuade doctors from offering the most suitable therapy options to their patients, regardless of price. Despite this, healthcare systems vary widely and the possibility for this to limit effective SDM cannot be discounted (92).

One might also consider the implications of healthcare payer restrictions. Grimberg et al. reported the consequences of insurance-mandated brand switches during paediatric treatment with GHT. Members of the Pediatric Endocrine Society were surveyed (*n* = 213) and asked to reflect on their treatment following treatment switching as determined by the healthcare payer. Most commonly cited concerns included errors in dosing and treatment lapses resulting from patients having to familiarise themselves with a new GHT (93). Treatment switches of this kind were also found to be associated with an increase in time for the endocrine team because of additional paperwork, educational needs, and reassurance of patients via the telephone. Together, this study indicated that insurance-mandated treatment switches may have a negative impact on patient care and contribute to an increased burden on endocrine staff resources. These healthcare-system challenges may also go some way to undermine opportunities for SDM given that patient choice is ultimately overruled by healthcare payer restrictions (93).

In a systematic review by Gravel et al. to determine commonly cited barriers to the implementation of SDM, challenges relating to the healthcare system that were identified also included a lack of staff to put SDM into practice (85), insufficient support from the healthcare organisation, inadequate access to the services needed to put SDM into place, and insufficient reimbursement for implementing SDM (85). Furthermore, a study investigating the way in which general practitioners (GPs) in the UK implemented the National Health Service (NHS) policies of greater patient involvement, identified a willingness to implement SDM but a disparity in its implementation given resource restrictions (59). Some GPs viewed SDM only as a means to improving “compliance” with treatment, rather than as an ethical consideration, while others understood the importance of SDM but felt there was disparity with its interpretation in practice. These GPs also recognised that enhanced SDM within the clinic required a greater level of training and consultation time, both of which have financial impact on NHS resourcing (59).

## Practical suggestions to overcome barriers to SDM in GHT

**Figure 2:**
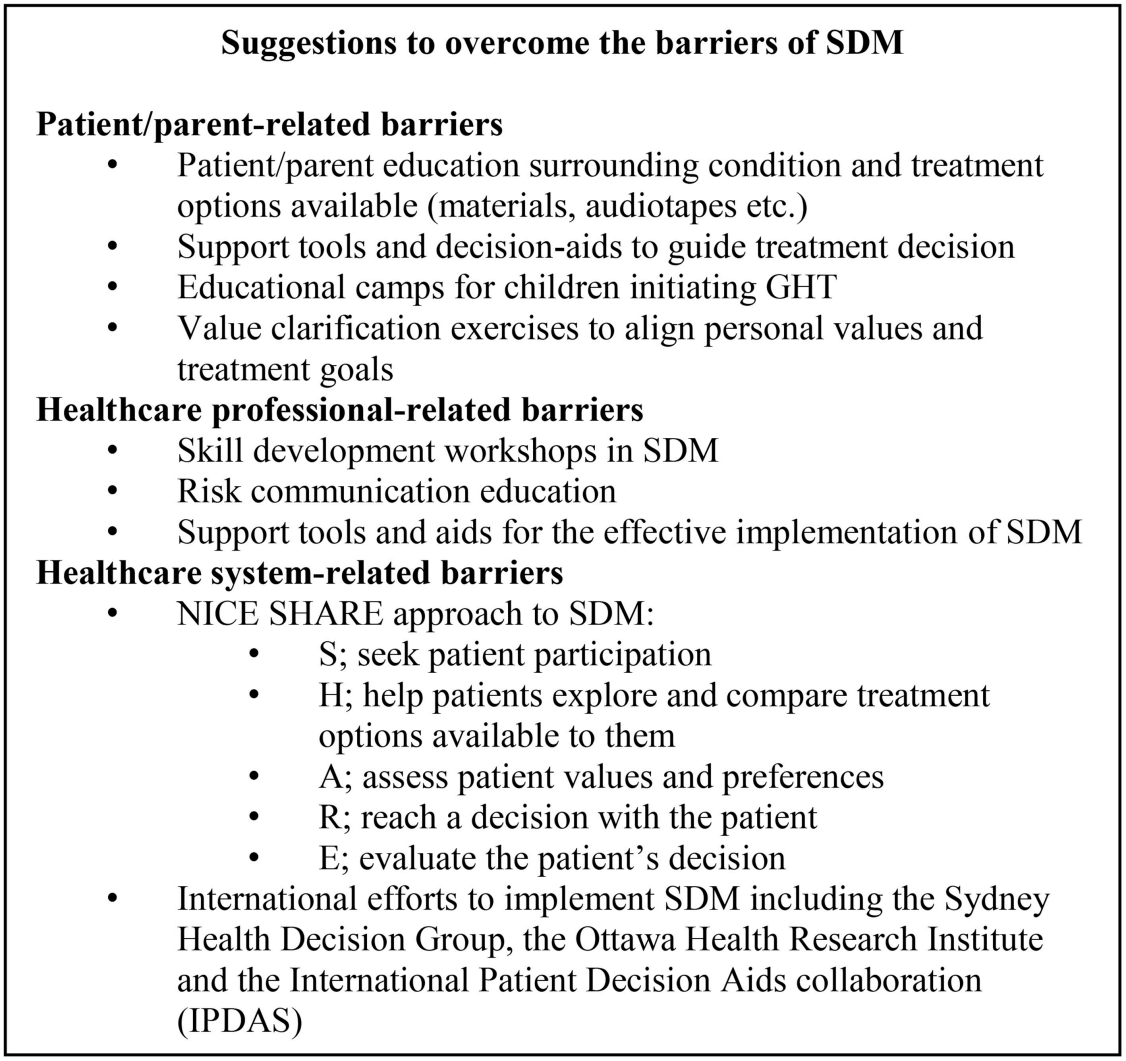
Solutions to overcome the barriers of SDM. GHT, growth hormone therapy; NICE, National Institute of Health and Care Excellence; SDM, shared decision-making.

There are several practical solutions that may be put in place to overcome some of the identified barriers to the effective implementation of SDM. Primarily, education of all parties involved in SDM is an important means by which patient involvement in their treatment decisions may be facilitated (Figure 2).

### Patient/parent-related barriers

As identified in section Barriers to the Implementation of SDM, patient/parent-related barriers to SDM highlight a critical need for patient education. A consensus statement on the management of GH-treated adolescents in the transition to adult care details the “education to ensure that patients have an understanding of their disease to develop autonomy in health care decision-making” (94). In line with this, appropriate education to better inform patient decisions (at all ages) is of crucial importance if SDM is to be effectively implemented and may go some way to also positively influence treatment adherence and persistence (45, 95).

As an example, a study by Lopez Siguero et al. reported a disparity between patient acceptance of their treatment and knowledge of their treatment when asked to complete a questionnaire. Higher questionnaire scores were achieved by patients who attended an education camp on GHT, highlighting the benefits of appropriate patient education (96). Education is thus important not only to enhance patient knowledge but also to encourage a greater ownership of their own treatment plan.

Gamification technologies may also support patient education by providing fun and interactive ways to teach patients about their disease and the benefits of GHT (97). Gaming technologies have been shown to promote self-management behaviour in children with type 1 diabetes. A smartphone app developed for patients records and rewards efforts to self-manage blood sugar measurements and has also been shown to improve physical activity in children (98). Additionally, application of virtual pet technology to GHT may also encourage patient education and promote self-management of their treatment. The virtual pet requires care each day and in doing so, establishes a “daily play moment” that may support adherence to GHT and may help to decrease the anxiety that may be associated with daily injections (97).

**Figure 3:**
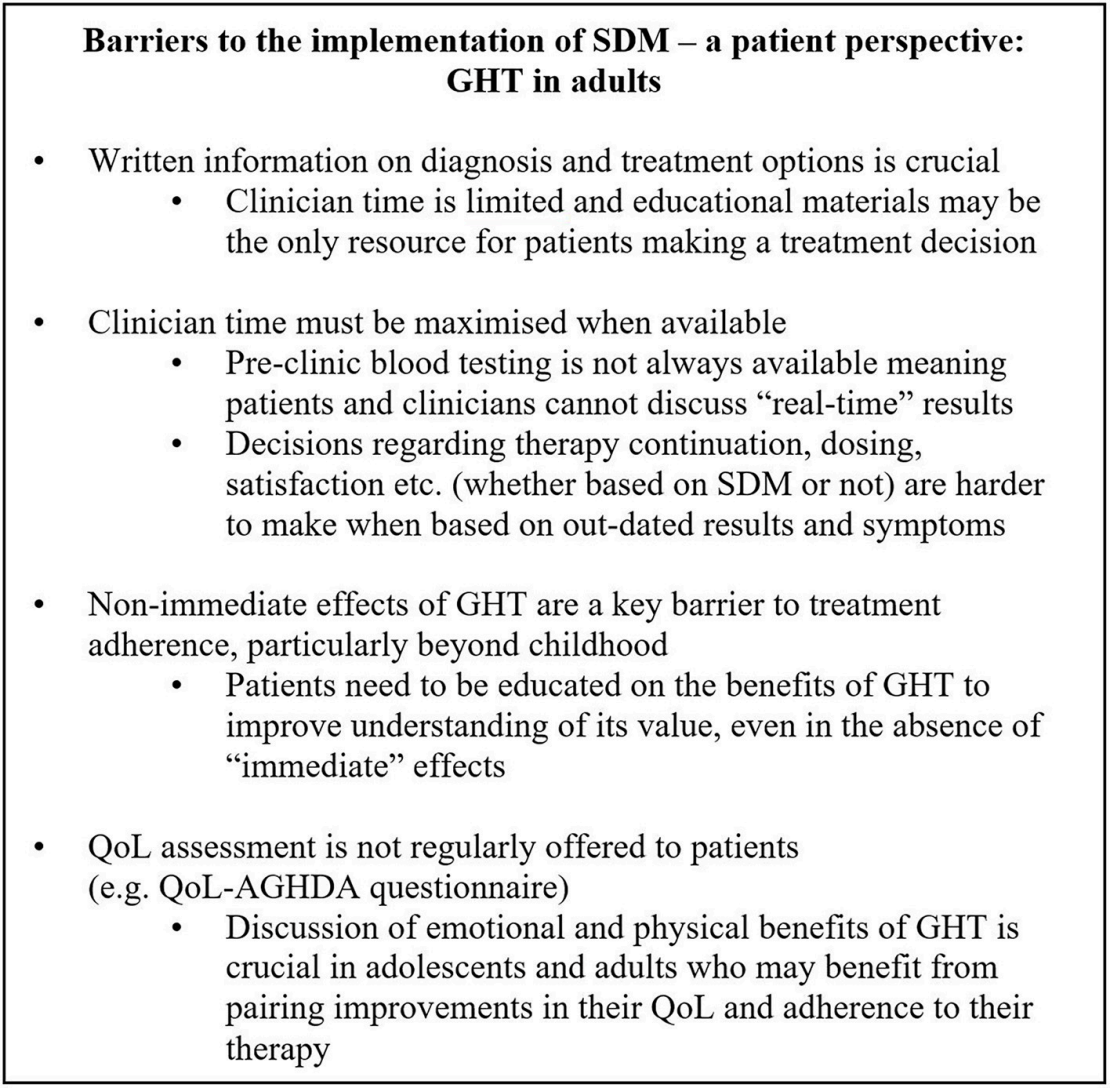
Barriers to the implementation of SDM—a patient perspective: GHT in adults. GHT, growth hormone therapy; IGF-1, Insulin-like growth factor-1; QoL, quality of life; QoL-AGHDA, Quality of life assessment of growth hormone deficiency in adults; SDM, shared decision-making.

Consideration of the non-growth-related consequences of GH-related disorders may also be key to engaging patients such that they are empowered to take an active role in their treatment journey, via the practice of SDM with their doctor. Whilst height velocity/linear growth are the most commonly assessed measures of treatment success in prepubertal patients, the benefits of GHT may extend beyond improved growth. GHT is known to have a role in body composition, lipid metabolism, androgen action, myocardial function, and cardiovascular health, as well as, quality-of-life perception (46). Indeed, education into the health implications of GH-related disorders, not limited to effects on growth must be clearly explained to patients and their families. A patient perspective of barriers to SDM in adult patients receiving GHT is detailed in Figure 3 and emphasises the need for adequate education of the non-immediate and non-growth implications of GHT. Similarly, in paediatric patients, levels of parent anxiety may differ when confronted with reduced height velocity as opposed to a serious GHD-related health issue and may consequently elicit different parent responses to treatment options.

Decision support tools are another popular tool used to aid patient/family education and decision-making. There are several examples of support tools that have proved effective in a variety of therapy areas. In a meta-analysis of 105 studies that investigated decision aid utility in 31,043 patients, decision support tools and aids were found to increase patient knowledge, awareness of risk factors associated with treatment and improve alignment between personal values and the final decision made (40).

In a study to evaluate a decision aid use in cardiovascular disease patients, patients were more encouraged to design an action plan for their treatment and to monitor their own progress. Indeed, 86–93% of patients rated the information in the decision aid as “excellent” or “very good” and resulted in an improved disease awareness and knowledge, as well as, a more coherent understanding of personal risk factors, treatment options and personal values (99).

**Figure 4:**
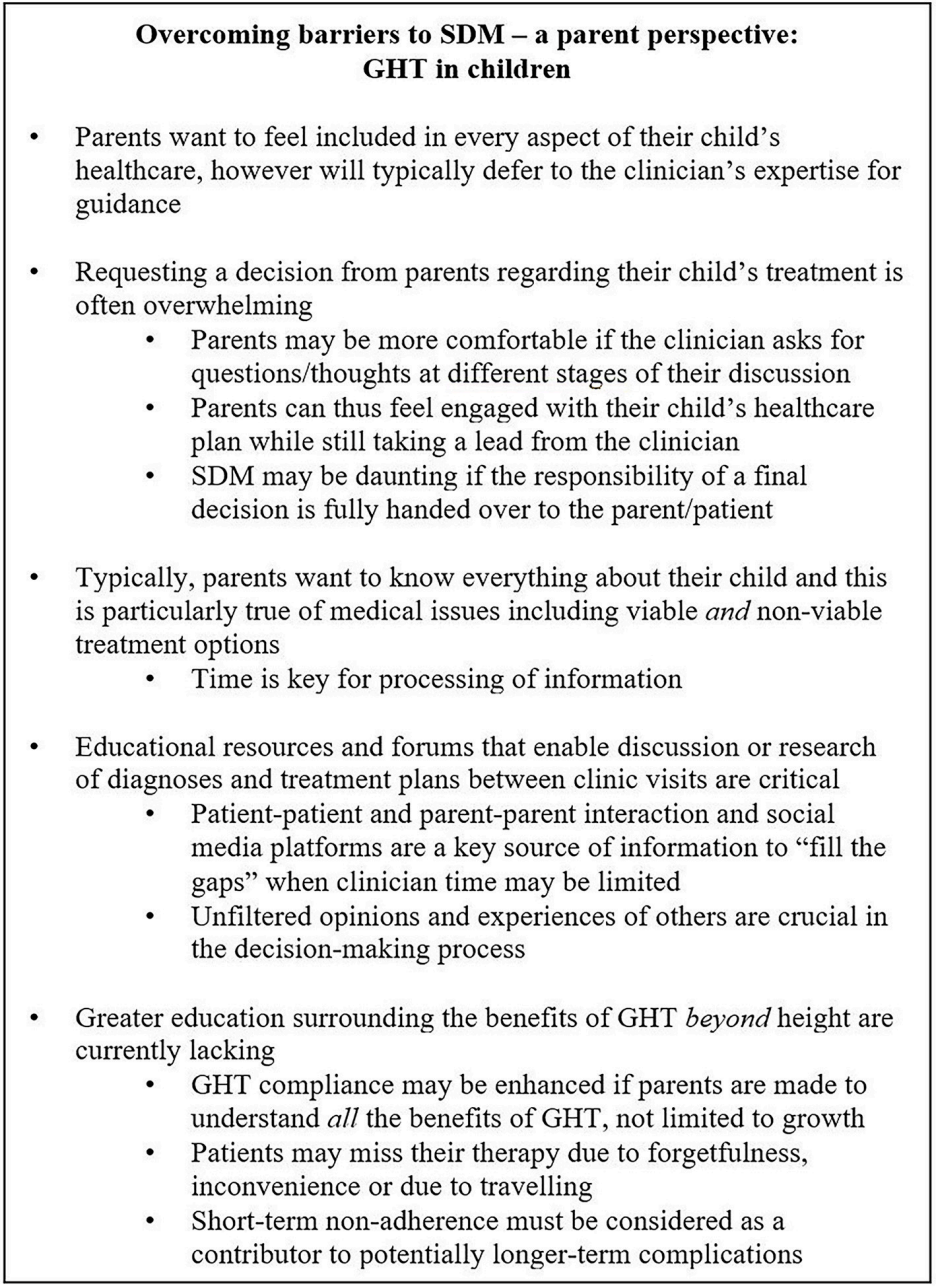
Overcoming barriers to SDM—a parent perspective: GHT in children. GHT, growth hormone therapy; SDM, shared decision-making.

Patient support groups may be another means by which SDM is supported within the clinic. Shared experiences between patients and their families may serve to enhance patient confidence in their treatment regimen and facilitate a supportive environment in which questions or concerns may be raised and answered. Support groups for GHT patients and families thus might help drive SDM within the clinic by providing an educational resource during times when face-to-face contact time with healthcare professionals is limited. Figure 4 details a parent-perspective of GHT in children and stresses the importance of effective patient and parent support groups between clinic visits.

Overall, strategies to support patient education and decision-making serve to better inform treatment plans and encourage patients to take a more active role in their treatment journey. While the evidence supporting the value of patient decision aids is largely reliant on collation of data collected from patient surveys and interviews (as dictated by the qualitative nature of the outcomes being assessed), there is a growing body of evidence to support the value of tools to allow patients to consider their personal values and treatment goals so as to create a more meaningful ownership of treatment and a confidence in the final decisions made. If patient empowerment is the key to treatment adherence, education is a crucial vehicle to achieving this.

### Healthcare professional-related barriers

With respect to healthcare-professional barriers, training activities to support clinicians and encourage the implementation of SDM may be appropriate. A study by Elwyn et al. evaluated the impact of skill development workshops for SDM and the use of risk communication in consultation with patients. This study reported that the level of clinician involvement in SDM was significantly increased by attendance of skill development workshops. Clinicians could acquire the skills they needed to more effectively introduce SDM within their clinic and engage patients in an active role in their treatment decisions. Ultimately, patient involvement may be better supported by the skill development of healthcare professionals in this area (100). Indeed, education of all parties (not limited to the patient) involved in SDM must be considered such that a well-informed decision may be mutually agreed upon.

### Healthcare system-related barriers

Healthcare system-barriers may be somewhat harder to overcome given the financial and resourcing restrictions of healthcare systems all over the world.

The Agency for Healthcare Research and Quality have established the SHARE approach—a model for SDM within the clinic (101). This five-step process aims to offer a means by which SDM can be implemented in the clinic: S, seek patient participation; H, help patients explore, and compare treatment options available to them; A, assess patient values and preferences; R, reach a decision with the patient, and E, evaluate the patient's decision. The SHARE approach has also established a workshop curriculum with resources to support the training of healthcare professionals and tools to support the implementation of SDM within the clinic. There are also several international groups and programmes in place to encourage the facilitation and implementation of SDM within clinics around the world. Many countries are involved in the International Patient Decision Aids collaboration and have established initiatives to support the implementation of SDM in clinical practice. Together, these collaborations are examples of healthcare systems supporting SDM and recognising its value in improving patient experience of care, and ultimately patient adherence to their treatment. One must however be aware of the challenges associated with a “one size fits all” approach and must consider the stages in which SDM may be most appropriate, given the subtleties of individual patients, case specifics and healthcare system rules.

Most important to note perhaps when considering the potential challenges to patient involvement, education is a modifiable barrier to SDM. Whilst healthcare resource restrictions and healthcare professional time may be harder to overcome, an effective implementation of SDM must recognise the opportunities for active patient involvement and seek to modify and/or alleviate those barriers that can be tackled given the confines/specifics of an individual patient case. Different patients treated in different healthcare systems may dictate a very different approach to SDM.

## Conclusions

In summary, adherence to GHT is not optimal and there are several reasons why patients may not fully adhere or persist with their treatment regimen. A move from a clinician paternalistic approach to a more patient autonomous approach to healthcare has seen a greater drive to implement SDM within the clinic in all therapy areas, not only GHT. SDM will not only ensure the implementation of the medical ethics principles of autonomy, beneficence, and non-maleficence, but also has been shown to improve adherence and treatment outcomes. The scope of SDM in the context of GHT is broad and there are several opportunities to implement SDM within a patient journey with GHT.

It is important to consider the barriers to the implementation of SDM and the ways one might overcome these challenges. Finding solutions to these challenges will enable patient empowerment and ownership of their own treatment plan and health goals. Most importantly, education is the primary means by which patient empowerment may be supported and must be provided to all decision-making parties including patients, their families and doctors. Strategies to improve education help to inform treatment decisions, and nurture patient confidence and satisfaction with that final decision; whatever it may be. Patient empowerment through education in this way will encourage improved treatment adherence, persistence and ultimately, enhanced treatment outcomes and success.

## Author contributions

CA, DS, SC, KT, NN, SR, and MM are responsible for the drafting and critical review of this manuscript during all stages of development and approved the final version for submission.

### Conflict of interest statement

CA has received research support and honoraria for lectures and participation in advisory boards from Novo Nordisk, Pfizer, Merck Serono and Ferring. SC has received honoraria for lectures and participation in advisory boards from Novo Nordisk, Sandoz, Pfizer and Merck Serono. MM has received research support and honoraria for lectures and consultancy from Ipsen, Merck Serono, Sandoz, Ferring, Pfizer, Lilly, and Novo Nordisk. NN and SR are employees of Novo Nordisk Health Care AG. DS and KT have received honoraria for participation in advisory boards from Novo Nordisk.
